# Accidental Aspiration of an Implant Driver Into the Right Bronchus During Implant Surgery

**DOI:** 10.7759/cureus.80955

**Published:** 2025-03-21

**Authors:** Ranjeet R Gandagule, Sayali Korde, Abhilasha Masih, Nikita Saini

**Affiliations:** 1 Department of Prosthodontics and Crown and Bridge, Dr. Hedgewar Smruti Rugna Seva Mandal's Dental College and Hospital, Hingoli, IND; 2 Department of Prosthodontics and Crown and Bridge, Sinhgad Dental College and Hospital, Pune, IND; 3 Department of Pedodontics and Preventive Dentistry, Hitkarini Dental College and Hospital, Jabalpur, IND

**Keywords:** aspiration, aspiration hazard, bronchoscopy, dental implant complications, dental practice management, emergency medical service, emergency protocol, implant driver

## Abstract

Accidental aspiration of dental instruments during implant surgery is a rare yet serious complication that demands immediate attention. This report presents a case involving a 67-year-old male who accidentally aspirated an implant driver during the placement of mandibular implants. Despite the absence of immediate symptoms such as coughing, choking, or wheezing, radiographic evaluation was crucial in identifying the foreign object. Thoracic and spinal X-rays, followed by computed tomography scans, confirmed its location in the right main bronchus. A thoracic surgeon successfully removed the implant driver using a rigid fiber-optic bronchoscope under general anesthesia. Postoperative care included monitoring in the intensive care unit with nebulization, antibiotics, and corticosteroids to prevent complications. This case highlights the importance of rapid diagnosis and intervention in such emergencies while emphasizing the need for preventive measures to mitigate aspiration risks during dental procedures.

## Introduction

A very rare but severe complication seen during dental procedures may be the ingestion or aspiration of a foreign object. In these cases, 87% were associated with instrument ingestion, while 13% occurred due to aspiration [1]. Objects that may be ingested or aspirated are impression material, endodontic files, orthodontic brackets, crowns, teeth, posts, burs, or implant components [2]. Implant procedure instruments are small, with implant components being even tinier. The presence of saliva can make these tools slippery, heightening the risk of them slipping from the operator’s grasp and potentially being ingested or aspirated by the patient [3]. Patients swallowing foreign bodies are usually asymptomatic initially, but the symptoms may develop later. Ingestion of a foreign object can cause damage to gastric mucosa, septic abscess, or intestinal perforations. Aspiration can lead to varying degrees of airway obstruction, ranging from partial to complete blockage, potentially resulting in post-obstructive pneumonia, respiratory distress, pneumothorax, or hemorrhage. Additionally, it may give rise to secondary infections such as aspiration pneumonitis and can cause unilateral lung collapse due to hypoventilation following bronchial obstruction [4-6]. Unintentional aspiration of dental appliances poses a greater risk than ingestion and should always be managed as a medical emergency. The employment of thin, pointed instruments enhances the susceptibility to perforation and pneumothorax [7].

At least one symptom from the typical triad of coughing, choking, and wheezing is observed in over 91% of patients with foreign body aspiration. Treatment necessitates bronchoscopy, and with expertise, foreign body removal can be straightforward, highly successful, and associated with minimal complications [8]. The possible complications of rigid bronchoscopy can be a mucosal tear, damage to the tracheal wall, tracheoesophageal fistula, mediastinitis, or hemorrhage. This clinical report describes the accidental aspiration of an implant driver into the right main bronchus and a rigid fiber-optic bronchoscope was used to retrieve it.

## Case presentation

**Figure 1:**
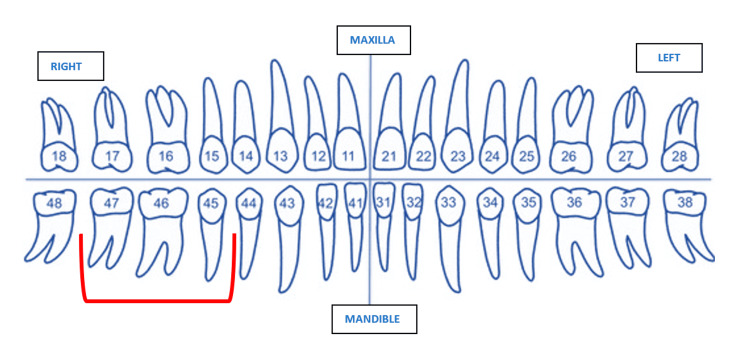
FDI (Fédération Dentaire Internationale) tooth numbering system showing missing teeth 45, 46, and 47 (marked in red) for which the patient sought replacement. Figure created by the author.

A 67-year-old man was referred to the department of prosthodontics and crown & bridge for the replacement of three missing teeth in the mandibular right posterior region (tooth numbers 45, 46, and 47) (Figure 1).

After evaluating the existing health condition, the patient was given treatment options of removable partial dentures and implants of which the patient opted for implants as a treatment of choice. During the surgery, the patient was positioned in a supine position on the dental chair, and three implants were placed in the mandibular right posterior region (tooth numbers 45, 46, and 47). During the tightening of the posterior-most implant (tooth number 47), the implant driver slipped from the fingers of the operator and was swallowed by the patient. The patient was immediately made to sit upright, and the Heimlich maneuver was performed to expel the implant driver. The implant driver could not be removed by the Heimlich maneuver and the patient was asymptomatic.

**Figure 2:**
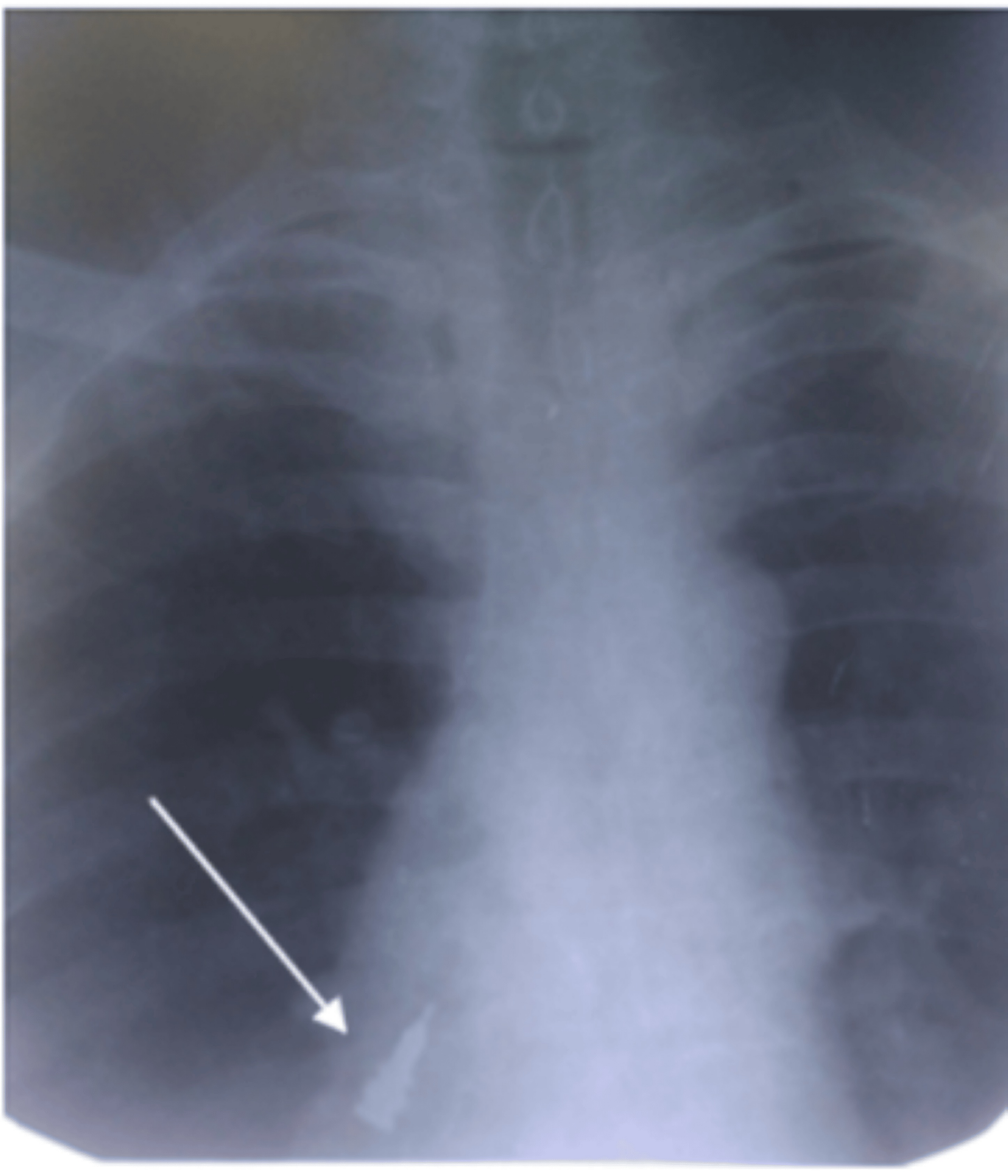
Thoracic and spine radiograph indicating a radiopaque foreign object.

Symptoms such as coughing, choking, and wheezing were absent. After reassuring the patient and placing a gauze pack at the site of the surgery, the patient was immediately taken to the department of oral radiology where thoracic and spine radiographs were made with the patient lying on his back in a supine position. A radiopaque foreign object was visible on the right side, but its exact location, whether in the lungs or the gastrointestinal tract, was uncertain (Figure 2).

**Figure 3:**
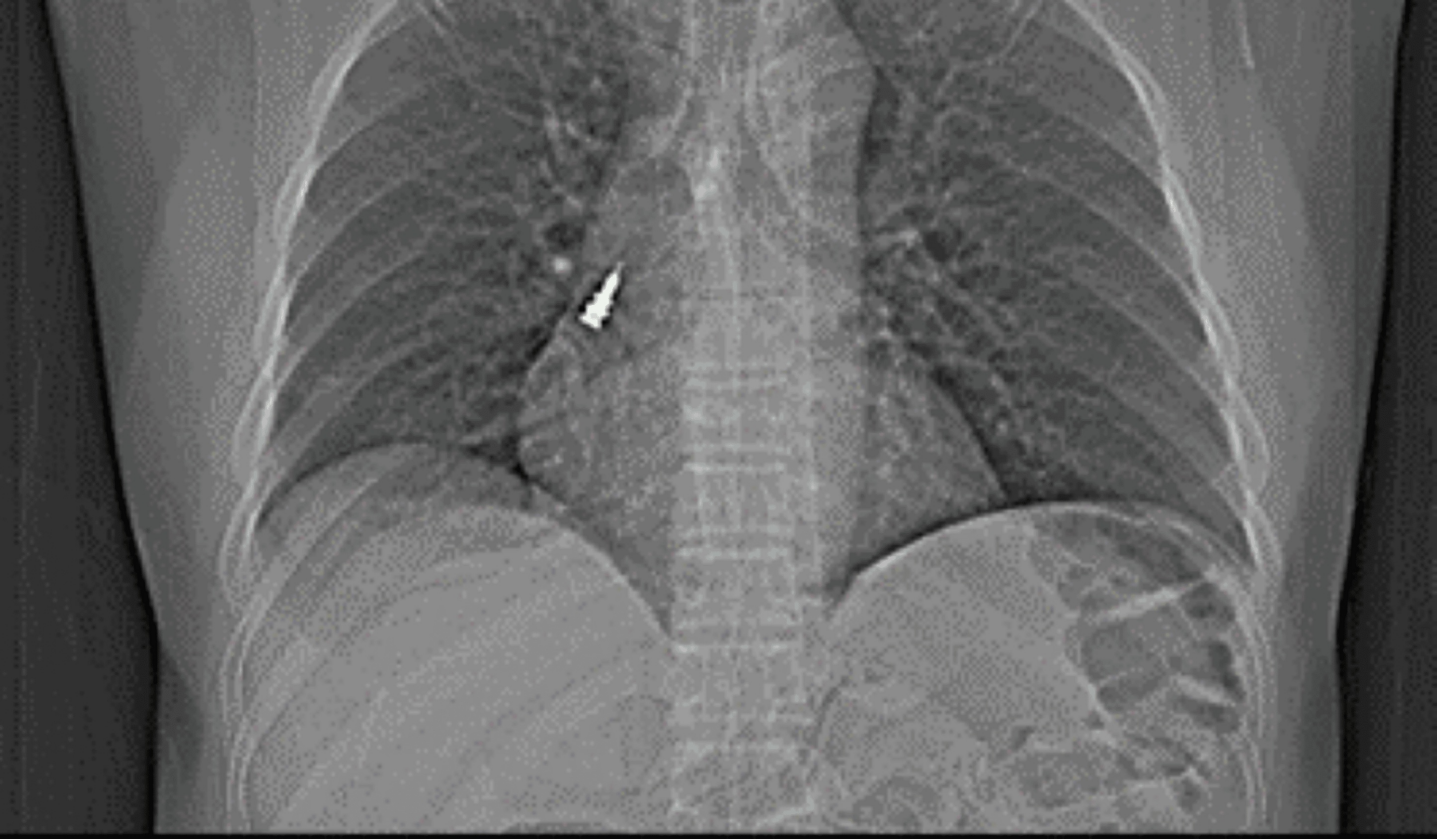
Chest X-ray confirming the presence of an implant driver in the right bronchus.

This radiopaque image was identified as the implant driver. The patient was immediately taken to the emergency ward at the medical hospital where further X-rays were made (Figure 3).

**Figure 4:**
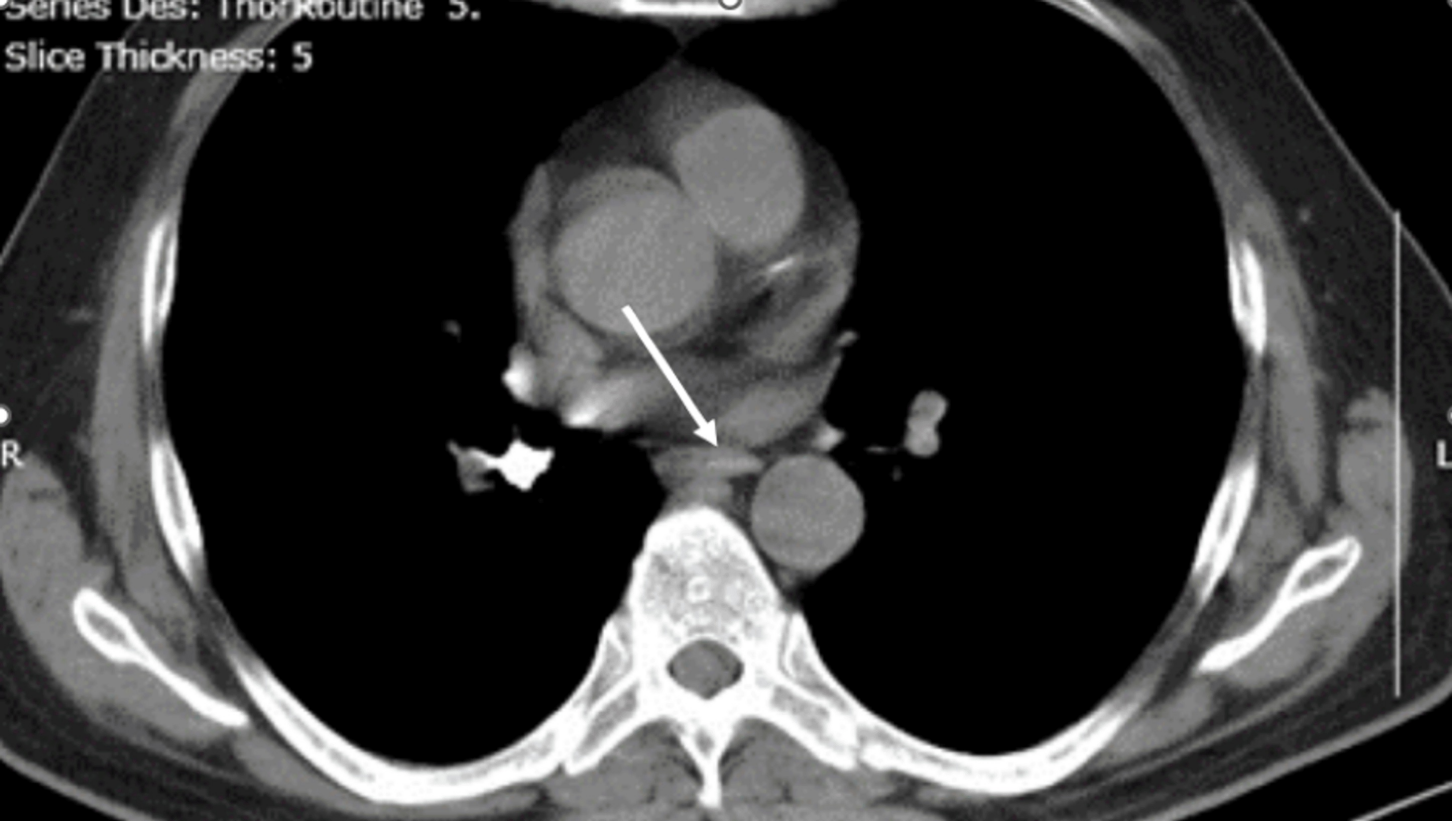
CT scan showing the impaction of the implant driver in the right main inferior bronchus.

CT scanning was also performed (Figure 4). The scanning reports showed the right main inferior bronchus with impaction of the radiopaque implant driver.

**Figure 5:**
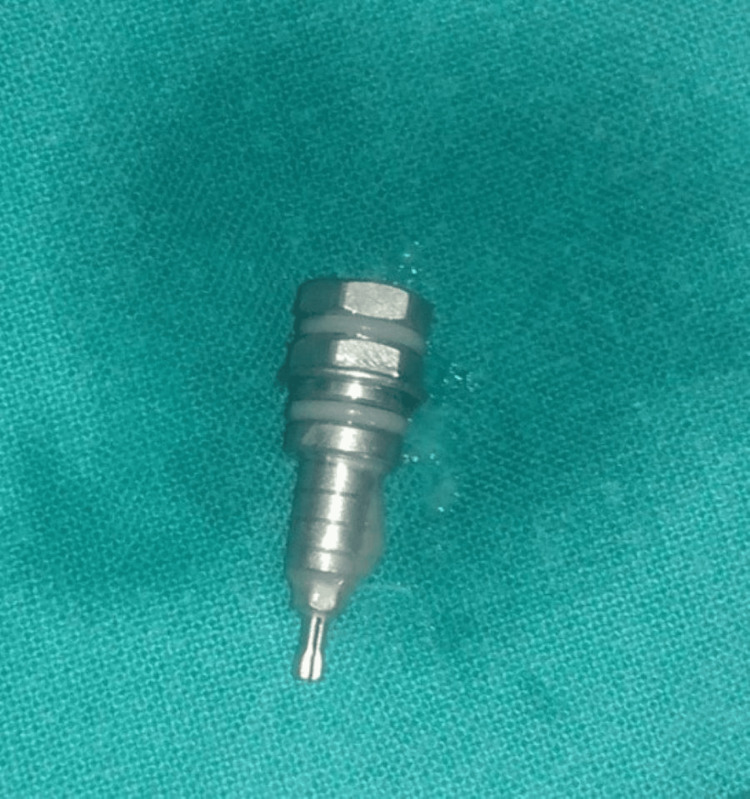
Retrieved implant driver following bronchoscopic removal.

Bronchoscopy was chosen as the preferred treatment method for removing the dental instrument. It was carried out by a thoracic surgeon using a rigid fiber-optic bronchoscope with a light source (Chevalier Jackson) with the patient under general anesthesia in the operating room. This method identified an endobronchial foreign body blocking the right main bronchus, which was successfully extracted during the procedure. After the removal of the foreign object, the patient was kept in the intensive care unit for 24 hours and his vital signs were monitored. (Figure 5).

Nebulization was done after the bronchoscopy procedure with Duolin (salbutamol and ipratropium) every six hours. He was administered injectable Taxim 500 mg twice a day for three days as a prophylactic antibiotic. To reduce inflammation, injectable dexamethasone 16 mg was administered every eight hours. The dose was tapered after three days. No further complications were observed. The patient remained stable and was discharged after the complete resolution of the episode.

## Discussion

Aspiration or ingestion of a foreign object is a rare but severe complication encountered in dental practice. The patient may be symptomatic or asymptomatic, therefore radiographs play a significant role in determining the presence and location of the foreign object. The sharp nature of the instrument heightens the risk of perforating anatomical structures [9,10]. Thus, as soon as the instrument exits the oropharynx, it is crucial to assess whether the foreign body has entered the gastrointestinal tract or the respiratory system [11]. Studies indicate that these iatrogenic errors are more commonly observed during the treatment of posterior mandibular teeth [1]. Among foreign bodies that enter the gastrointestinal tract, 80% to 90% pass naturally, 10% to 20% necessitate nonoperative intervention, and fewer than 1% require surgical removal [12-15]. In adults, aspirated foreign bodies are more likely to obstruct the right bronchial system. Removal is typically performed using either flexible or rigid bronchoscopy [16].

In this clinical report, the patient who was undergoing treatment for the placement of implants, accidentally aspirated an implant driver while tightening the posterior-most, right mandibular implant (tooth number 47). The patient was asymptomatic and did not present any symptoms from the triad of choking, coughing, or wheezing. If a foreign object enters the distal bronchial system without immediately causing a blockage, it may go unnoticed for some time, depending on its characteristics. Patients who inhale small inorganic materials often remain asymptomatic for an extended period unless a distal airway becomes completely obstructed. Thoracic and spinal X-rays, along with computed tomography scans, assisted in identifying the presence and location of the implant driver within the right bronchus. A rigid fiber-optic fibroscope was used by the thoracic surgeon for the removal of the implant driver [17].

Not many case reports are described for aspiration of a foreign object because it is a rare incident. This accident could have been prevented if the implant driver had been tethered, if a rubber dam or gauze screen had been used to cover the oropharynx, or if the patient had been operated in a semi-reclined position.

## Conclusions

Aspiration of dental instruments is a potentially life-threatening event that requires immediate recognition and appropriate management. This case underscores the importance of early radiographic evaluation and bronchoscopy in confirming and retrieving aspirated foreign objects. The absence of initial symptoms should not delay intervention, as undiagnosed foreign-body aspiration can lead to severe complications, including airway obstruction, pneumonia, or lung collapse. Implementing preventive strategies, such as using gauze screens, rubber dams, or tethered instruments, is essential to minimize such risks. Additionally, practitioners should maintain a high level of vigilance during procedures, particularly when working in the posterior regions of the oral cavity. In case of aspiration, prompt referral to a medical emergency facility is critical to ensuring optimal patient outcomes.
